# Neutrophil-to-Lymphocyte Ratio in the Alzheimer’s Disease Continuum

**DOI:** 10.3390/ijms26115157

**Published:** 2025-05-28

**Authors:** Davide Aprile, Fabiola De Marchi, Federico Menegon, Cristoforo Comi, Giacomo Tondo

**Affiliations:** Neurology Unit, Department of Translational Medicine, Università del Piemonte Orientale (UPO), 28100 Novara, Italy; davide.aprile@uniupo.it (D.A.); fabiola.demarchi@uniupo.it (F.D.M.); federico.menegon@gmail.com (F.M.); giacomo.tondo85@gmail.com (G.T.)

**Keywords:** neuroinflammation, mild cognitive impairment, biomarkers, cognitive decline

## Abstract

Alzheimer’s disease (AD) is a neurodegenerative disorder defined clinically by progressive cognitive decline and memory impairment and pathologically by the accumulation of amyloid-beta plaques, tau neurofibrillary tangles, neuroinflammation, and immune system dysregulation. Peripheral biomarkers are gaining attention as valuable tools for elucidating neuroinflammatory mechanisms in the AD continuum, with potential implications for diagnosis and prognosis. Among these, the neutrophil-to-lymphocyte ratio (NLR) has emerged as a promising systemic inflammatory marker. NLR, a readily available and cost-effective parameter derived from routine blood tests, reflects the balance between innate and adaptive immune responses. Elevated NLR has been associated with AD and mild cognitive impairment (MCI), showing correlations with disease severity, amyloid burden, and neuroinflammation. Increased neutrophil counts may contribute to neurodegeneration through oxidative stress and pro-inflammatory cytokine release, while decreased lymphocyte levels suggest impaired adaptive immunity. However, despite growing evidence, the clinical utility of NLR in AD remains debated due to heterogeneity in study populations and confounding factors, such as comorbidities and medication effects. This review provides a comprehensive analysis of the association between NLR and AD throughout the disease continuum. Future research should prioritize longitudinal studies and integrative approaches that combine NLR with other inflammatory and neurodegenerative markers to enhance early diagnosis and personalized therapeutic strategies.

## 1. Introduction

Alzheimer’s disease (AD) is the most common neurodegenerative disorder and the leading cause of dementia. AD-related dementia is characterized by memory impairment and deficits in other cognitive domains, ultimately affecting the ability to perform activities of daily living. Most AD cases occur in individuals over 65 years of age, as the prevalence of AD increases significantly with aging [1,2]. Mild cognitive impairment (MCI) represents an intermediate stage between normal cognitive function and AD dementia, identifying a population at risk for developing AD [3]. However, not every MCI case converts to dementia, and some patients may remain stable or revert to normal cognition. Identifying markers of pathology or neurodegeneration is particularly important in the MCI condition, when prognostic prediction becomes crucial to favor a multidisciplinary approach able to minimize the exposure to risk factors and prevent conversion [4].

**Figure 1:**
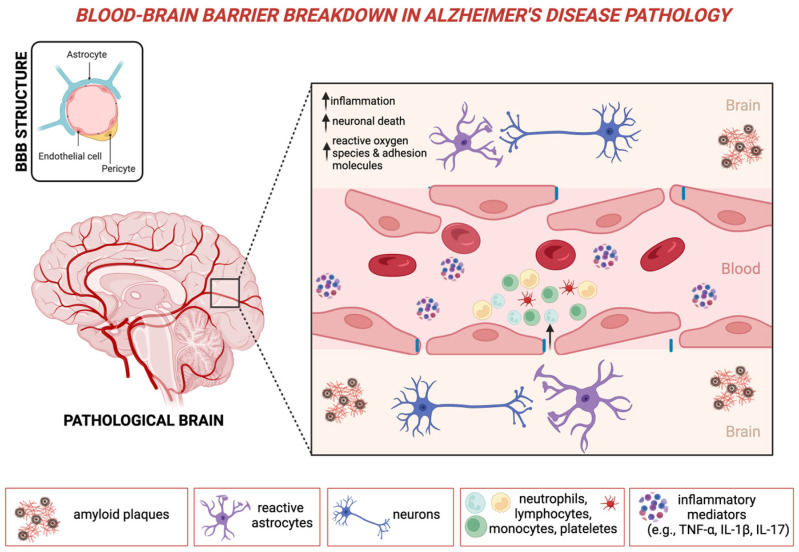
Blood–brain barrier breakdown in Alzheimer’s disease pathology. This schematic representation illustrates the involvement of NLR in AD, emphasizing its contribution to neuroinflammation and BBB dysfunction. The pathological brain in AD is characterized by amyloid plaque accumulation and neurofibrillary tangles, leading to neuronal damage and activation of reactive astrocytes. Increased NLR reflects a systemic inflammatory response, with elevated neutrophils contributing to oxidative stress, cytokine release, and endothelial dysfunction, ultimately promoting BBB breakdown. The compromised BBB allows peripheral immune cells, including neutrophils, monocytes, and platelets, to infiltrate the brain parenchyma, exacerbating neurodegeneration. These processes collectively drive chronic neuroinflammation, neuronal death, and cognitive decline in AD. Abbreviations: BBB: blood–brain barrier; IL: interleukin; TNF: tumor necrosis factor. Created with Biorender.com (De Marchi F.).

A large body of literature has demonstrated that the pathogenesis of AD involves multiple mechanisms, including the accumulation of amyloid-beta (Aβ) and tau proteins, activation of neuroinflammatory pathways, disruption of the blood–brain barrier (BBB), and neurovascular dysfunction [5]. These processes, summarized in Figure 1, collectively contribute to neurodegeneration and begin many years before the onset of clinical symptoms, often preceding a formal diagnosis by decades [6]. AD’s hallmarks are Aβ plaques and tau tangles, but additional mechanisms also drive neurodegeneration. This complexity partly explains why therapies targeting Aβ and tau have largely failed [7,8]. Neuroinflammation contributes to Alzheimer’s by linking chronic inflammation to protein buildup and neuronal damage. However, the sequence of events remains unclear, with both persistent inflammation and failed protective mechanisms likely playing a role [9,10,11].

In vivo and human studies have reported elevated levels of pro-inflammatory and anti-inflammatory cytokines in the cerebrospinal fluid (CSF) of individuals with AD. These molecules, including interleukins (ILs) such as IL-1β, IL-4, IL-6, IL-9, and IL-17A, correlate with cognitive decline and disease progression, suggesting that a dysregulated inflammatory environment may affect cognition [12]. In addition, alterations of inflammatory markers can be detected along the entire AD continuum, since transforming growth factor β (TGF-β), IL-7, intercellular adhesion molecule-1 (ICAM-1), and tumor necrosis factor α (TNF-α) have been associated with clinically meaningful cognitive decline over a 1-year follow-up period [13]. Most studies have focused on the role of microglia and increased activation of microglia, the brain’s primary innate immune cells. In humans, PET imaging of the translocator protein (TSPO) reveals neuroinflammatory responses in vivo by detecting microglia activation, and it has been widely used in AD [14]. A certain degree of microglial activation has been observed not only in patients with AD but also in individuals with MCI [15,16,17,18]. These findings suggest that neuroinflammation plays a key role in early neurodegeneration, with multiple microglial phenotypes driving a dysregulated response that promotes protein accumulation and accelerates disease progression [19,20]. Impaired clearance of protein aggregates, involving dysfunction of the glymphatic system, contributes early to AD pathology by promoting toxic protein accumulation and disrupting brain homeostasis [21]. In this context, the BBB regulates immune traffic into the central nervous system (CNS), maintaining brain immune privilege. In aging and neurodegeneration, BBB integrity weakens, allowing pro-inflammatory cells and molecules to infiltrate the brain. This breakdown facilitates abnormal communication between the peripheral and central immune systems, promoting a neuroinflammatory environment that may contribute to the progression of neurodegeneration [22].

The neutrophil-to-lymphocyte ratio (NLR) is a simple, accessible clinical index derived from routine peripheral blood tests, calculated by dividing the number of neutrophils by the number of lymphocytes. It is considered a marker of systemic inflammation and reflects the balance between the innate (neutrophils) and adaptive (lymphocytes) immune responses [23]. This review aims to comprehensively evaluate the existing literature regarding the NLR in the field of AD and MCI, examining the difference between AD and MCI subjects and healthy controls (HC), and exploring the diagnostic, prognostic, and staging value of the NLR along the AD continuum.

## 2. Peripheral Inflammation as a Primary Driver in Alzheimer’s Disease

Chronic systemic inflammation and immune activation are increasingly recognized as key mechanisms in the pathogenesis of AD. Recent studies have identified various peripheral inflammatory markers, such as neutrophil and lymphocyte counts, as well as their ratios, as reliable indicators of altered systemic immune status [22]. These markers may also reflect dysregulated immune responses occurring within the CNS. Both neutrophils and lymphocytes have been extensively studied in the context of AD, partly due to the ease with which their counts can be obtained and partly because they play central roles in mediating inflammatory responses. Moreover, they are considered potentially accessible and modifiable therapeutic targets, further supporting interest in their role in AD pathology [24].

### 2.1. The Role of Neutrophils in Neuroinflammatory Responses

Neutrophils are generally regarded as innate immunity markers, whereas lymphocytes are associated with adaptive immune responses. Alterations in ratios, including the NLR, may indicate an imbalance between these immune branches. Additionally, ratios—rather than absolute cell counts—can help reduce inter-individual variability and provide a more stable measure of systemic immune status [25]. Neutrophils are considered critical players in innate immunity due to their role in combating pathogens through mechanisms such as phagocytosis and the release of antimicrobial molecules [26]. However, these same mechanisms can become harmful when chronically activated, leading to sustained and uncontrolled inflammation. This prolonged inflammatory state is associated with tissue damage, excessive production of reactive oxygen species (ROS), and broader immune dysregulation, all of which may contribute to developing autoimmune diseases [27].

Evidence elucidating the role of neutrophils in AD comes from studies using transgenic mouse models. These include models such as 5xFAD and 3xTg-AD mice, which carry human mutations that promote AD pathology through mechanisms like Aβ plaque deposition (e.g., in APP/PS1 and 5xFAD) or combined amyloid and tau pathology (as seen in 3xTg-AD) [28]. These models have been invaluable for investigating how immune cells, including neutrophils, contribute to disease progression [29]. In AD mouse models, neutrophils exhibit increased activation, with enhanced migration across the BBB and accumulation in key brain regions such as the cortex and hippocampus. This leads to chronic vascular permeability, persistent inflammatory stimulation, and BBB disruption [30,31,32]. A potentially critical mechanism involves neutrophil adhesion to the endothelium and subsequent migration across the BBB, which drives chronic neuroinflammation. In the APP/PS1 murine model, neutrophils localized in the brain demonstrated increased adhesion to small blood vessels and degranulation around Aβ plaques, suggesting a mechanism of neuronal injury linked to vascular damage and oxidative stress [33]. Importantly, AD mouse models with induced deficiency of myeloperoxidase (MPO), a surrogate marker of neutrophil activity, showed significantly improved performance, particularly in spatial learning and memory tasks, compared to mice without MPO deficiency. These MPO-deficient mice also exhibited lower levels of neuroinflammatory markers [34].

Building on evidence from animal models, dysregulation of neutrophil activity has also been confirmed in patients with AD. Across several studies, transcriptional analyses of brain tissue have demonstrated a general increase in proinflammatory pathways involving neutrophils [35], exhibiting heightened activity, enhanced adhesion, and increased cell-surface interactions [36]. In AD brains, neutrophils have been identified in specific regions—including the temporal cortex and hippocampus—particularly near Aβ plaques, close to sites of BBB disruption, and in association with vascular dysfunction and increased cellular adhesion [24,33,37]. BBB breakdown is a key event that promotes neurodegenerative changes in AD and contributes to clinical cognitive decline. A large study involving individuals without cognitive impairment, as well as those with MCI or early dementia, found that BBB disruption was a risk factor for cognitive decline. Moreover, several inflammatory markers, such as ICAM-1, vascular endothelial growth factor (VEGF), and IL-8, which are involved in cell adhesion, neutrophil migration, and vascular remodeling, were associated with both BBB impairment and more rapid cognitive deterioration [38]. In addition, circulating neutrophils may contribute to neurodegenerative changes by differentiating into pro-inflammatory subtypes and promoting systemic chronic inflammation. Neutrophil hyperactivation has been reported in patients with AD, with these hyperreactive neutrophils producing elevated levels of ROS and correlating with faster cognitive decline. This suggests that neutrophil phenotype could serve as a prognostic peripheral marker in patients with dementia [39].

### 2.2. The Role of Lymphocytes in Neuroinflammatory Responses

Lymphocytes are a heterogeneous population of immune cells with various roles and specializations. As a key component of the adaptive immune system, they recognize and respond to specific antigens. Broadly, lymphocytes can be classified into T, B, and natural killer cells. In particular, T cells can infiltrate the CNS or act peripherally to influence brain pathology in neurodegenerative diseases [40]. Among T cells, several specialized subtypes play distinct roles in immune regulation. CD4+ helper T cells (Th cells) coordinate immune responses by secreting cytokines; however, they can also contribute to neurodegenerative processes by releasing proinflammatory cytokines, activating microglia, and disrupting the BBB [41]. The Th1 subtype is primarily involved in cell-mediated immunity, promoting inflammation through interferon-gamma (IFN-γ) secretion, while Th17 cells produce IL-17 and are implicated in autoimmune and neuroinflammatory responses [42]. Regulatory T cells (Tregs) are another crucial T-cell subtype, essential for maintaining immune tolerance and preventing autoimmunity. While CD4+ T cells are primarily involved in modulating neuroinflammation, CD8+ T cells also play a significant role in neurodegenerative diseases. Notably, CD8+ T cells infiltrate the brain parenchyma in patients with AD, showing clonal expansion in response to Aβ plaque deposition and BBB disruption [43]. Under physiological conditions, lymphocytes are unable to cross the BBB. However, in neurodegenerative diseases, particularly AD, the BBB becomes significantly more permeable, allowing lymphocyte infiltration into the CNS. CD4+ and CD8+ T lymphocytes have been detected in the brains of patients with AD. Moreover, dysregulation of lymphocyte subpopulations has been associated with AD pathogenesis and cognitive decline. CD4+ and CD8+ T cells, along with Th1, Th17, and Tregs, have been proposed to promote or modulate neuroinflammation in AD [44].

Animal models have further supported this hypothesis. In mouse models, hippocampal injection of Aβ to induce AD-related changes leads to BBB disruption and Th17 cell infiltration, accompanied by the overexpression of proinflammatory cytokines such as IL-17 and IL-22, contributing to neurodegeneration [45]. IL-17 appears to be mainly involved in AD-related pathology, as its presence is associated with exacerbated cognitive decline and synaptic dysfunction [46], while blocking IL-17 has been shown to reduce Aβ pathology [47]. Other immune mechanisms are also implicated. For example, in the APP/PS1 mouse model, infiltration of CD8+ T cells into the brain has been shown to impair synaptic plasticity and directly contribute to neuronal dysfunction [48]. Additionally, the depletion of Tregs in APP/PS1 mice accelerates cognitive decline, possibly by reducing early microglial activation, which may initially have a protective effect by promoting amyloid clearance [49].

The relationship between lymphocyte populations and AD pathogenesis has also been investigated in humans. An increase in Th17 cells has been observed in individuals with MCI, while altered Treg activity has been linked to tau protein deposition [50]. In patients with AD, reduced levels of circulating CD8+ T lymphocytes have been repeatedly reported [51], although CD8+ T cell infiltration into brain tissue has also been demonstrated [52]. Overall, an altered CD4+/CD8+ T cell ratio has been noted in AD, which may reflect either a relative increase in CD4+ cells or a decrease in CD8+ cells [51].

### 2.3. Peripheral Immune Dysregulation and the NLR

The above-mentioned findings suggest that both the innate and adaptive immune systems play a crucial role in the pathogenesis of AD and that an imbalance between them may contribute to neurodegenerative changes. Using ratios, such as the NLR, instead of absolute neutrophil or lymphocyte counts can help control for inter-individual variation in total cell counts. NLR, which was first described by Zahorec and colleagues two decades ago [23], has been proposed as a potential biomarker in a variety of medical conditions, including sepsis, stroke, cancer, and critical illness [53,54,55,56,57]. Neutrophils have become key contributors to inflammation, cancer progression, and cellular proliferation [58,59]. Conversely, a reduction in the number or function of Tregs has been linked to a decreased capacity to suppress inflammation [60]. Given its ability to reflect dysregulation in the peripheral immune system, the NLR has been widely investigated in neurodegenerative diseases, including AD, Parkinson’s disease, amyotrophic lateral sclerosis, and multiple sclerosis [61,62,63,64,65,66,67,68].

## 3. Neutrophil-to-Lymphocyte Ratio in the Alzheimer’s Disease Spectrum

The first approach to using the NLR in patients with neurodegenerative diseases, including AD, is to identify the specific population affected by neurodegeneration compared to the general population. This allows for assessing how specific and sensitive the index is in detecting the disease by comparing patients with HCs [51,61].

Higher values of the NLR have been consistently reported in patients with AD compared to the normal population, beginning with the first report over a decade ago [69]. The work of Kuyumcu and colleagues reported significantly higher NLR values in Patients with AD compared to HCs, proposing a cut-off value of 2.48, with approximately 70% sensitivity and 80% specificity for identifying Patients with AD. Subsequent research has provided further confirmation of these findings. Indeed, a higher NLR has been associated with AD in comparison to HC in several epidemiological studies [70,71,72,73,74,75]. Furthermore, increased NLR values have been hypothesized to be specific to AD dementia. The study by Cervellati and colleagues included patients with AD, vascular dementia, and MCI and HCs, and AD and MCI patients had higher NLR values than HCs, while no differences were observed between vascular dementia patients and HCs. This was the first study to report that NLR is significantly elevated in MCI and AD, but not in other dementia conditions [76]. Nevertheless, another study that investigated differences in NLR across dementia subtypes, including AD, vascular dementia, mixed dementia, frontotemporal dementia, and Korsakoff syndrome, as well as among depressed and non-depressed individuals, failed to identify statistically significant differences in white blood cell counts and ratios between groups [77].

While most studies support the observation of elevated NLR levels in Patients with AD relative to HCs, some studies have reported opposite or inconsistent findings [78]. The conflicting results may be attributed to several factors, primarily that peripheral blood counts, and consequently the derived indices, can be influenced by numerous variables. Moreover, neurodegenerative dementias are often highly heterogeneous conditions, even in the presence of biomarker evidence of AD [79]. NLR can be affected by demographic and clinical factors such as comorbidities, age, and sex. Comorbidities may influence cognitive decline by promoting pathological mechanisms that contribute to the development of dementia [80]. In turn, comorbid conditions can alter peripheral blood counts and inflammatory ratios [81,82]. Several studies exploring the effect of NLR have either corrected for the presence of comorbidities, excluded individuals with systemic inflammatory disorders, or omitted those taking medications that can alter peripheral blood counts, such as chronic steroid use [83]. Others have corrected for demographic, clinical, or genetic variables [70,84]. Due to these criticisms, the diagnostic utility of NLR in distinguishing Patients with AD from HCs has been questioned, as it may be significantly affected by confounding variables including age, sex, and the apolipoprotein E gene (APOE) allele status [70].

The condition of MCI is particularly interesting in biomarker research, as it provides an ideal framework for studying neurodegenerative dementias. MCI represents an intermediate state between normal aging and dementia, characterized by a high risk of progression to dementia, particularly AD. However, not all individuals with MCI progress to dementia; a substantial proportion remains stable over many years, and a small subset may even revert to normal cognitive and neuropsychological functioning [85]. Identifying risk factors associated with conversion to dementia is thus essential to enable early intervention, ideally before neurodegeneration becomes irreversible. Accordingly, the diagnostic utility of the NLR has also been proposed in the context of MCI, although with weaker results than those observed in AD. In 2019, Dong and colleagues compared routine peripheral blood parameters, including the NLR, among AD, MCI, and HC. The NLR was the only parameter that allowed discrimination of both MCI and AD from HC, with a proposed cut-off of 2.61 for AD and 2.25 for MCI. However, NLR values did not differentiate AD and MCI [72]. These findings have been reported in other similar studies. Kalelioglu and colleagues examined the NLR across four groups: HCs and individuals with subjective cognitive decline, MCI, and AD. The authors reported significantly higher NLR values in both the AD and MCI groups than in HCs, with mean values of 2.38 for AD and 2.48 for MCI, again without statistically significant differences between the AD and MCI groups [71]. Elevated NLR values in individuals with MCI compared to cognitively normal individuals have also been observed in other studies, suggesting NLR as a potential tool for identifying the prodromal stage of dementia [86]. Moreover, the NLR has shown potential for identifying MCI in specific populations, such as individuals with diabetes mellitus (DM). In a comparison between patients with DM and MCI versus those with DM but no cognitive impairment, the former group was older and exhibited higher NLR values. Additionally, patients with DM and MCI had lower scores on the Montreal Cognitive Assessment, which were negatively correlated with NLR levels. These findings suggested that the NLR may serve as an independent risk factor for MCI in patients with DM [87].

Fewer studies have explored the prognostic value of the NLR in the prodromal stage of dementia. Since MCI, particularly the amnestic subtype, is at risk of progressing to AD dementia, some studies have investigated the NLR’s ability to predict conversion. Using the “index of progression”, an indicator representing the number of points lost per year on the Mini-Mental State Examination (MMSE), a study on a large population of individuals with amnestic MCI demonstrated a direct correlation between cognitive decline over a two- to five-year follow-up period and several peripheral biomarkers, including neutrophil count, NLR, platelet-to-lymphocyte ratio, and the systemic immune-inflammation index (calculated as platelet count × neutrophil count/lymphocyte count). Furthermore, among several independent variables potentially associated with conversion, only the NLR and baseline MMSE score were significantly linked to a higher risk of conversion to dementia at follow-up. Lastly, individuals with MCI who exhibited higher NLR levels experienced significantly poorer outcomes, characterized by either a higher conversion rate to dementia or a more rapid cognitive decline over time [83]. Nevertheless, despite providing evidence for higher NLR values in individuals with amnestic MCI compared to HC, and a negative correlation between NLR and MMSE scores, another study found no significant differences in NLR between stable and progressing amnestic MCI groups [76].

To test the efficacy of a biomarker in identifying a disease process, revealing disease-associated pathways, or predicting disease progression, studies involving large populations assessed longitudinally over long follow-up periods are essential. NLR has been hypothesized as a potential tool for predicting conversion, identifying healthy individuals at risk of developing dementia, or serving as a candidate risk factor for dementia onset [88,89]. In the study by Ramos-Cejudo and colleagues, which was based on a large cohort from the extensive community-based Framingham Heart Study, NLR was independently associated with incident dementia over a follow-up period of approximately six years. Notably, NLR demonstrated predictive value second only to age, a well-established risk factor for dementia, alongside baseline cardiovascular disease and hypertension [89]. An increased risk of dementia in the general population linked to an imbalance between innate and adaptive immunity has been supported by other studies using various cellular counts and indices, such as granulocyte count [90]. Further evidence supports that increased innate immunity markers, including neutrophil count and NLR, can be associated with the development of cognitive impairment and dementia even after adjusting for demographic and genetic variables such as age, sex, education, body mass index, APOE, and ethnicity [84,91].

Similarly, studies analyzing large cohorts from public databases are valuable for confirming the correlation between NLR and AD-related changes and clarifying the potential role of NLR in staging disease severity and predicting cognitive decline. It has been suggested that the correlation between NLR and cognitive decline may be mediated by neurodegenerative changes such as brain atrophy [92,93], or by AD-related pathological changes such as Aβ and tau pathology [25,94]. Some studies have investigated these associations by analyzing multiple biomarkers using data from the Alzheimer’s Disease Neuroimaging Initiative (ADNI) [25,92,95]. In the study by Mehta and colleagues, NLR levels were higher in individuals with MCI and AD than in cognitively normal controls and were significantly associated with Aβ pathology as measured by amyloid-PET. These findings suggest that higher NLR is associated with greater Aβ deposition in the brain and more pronounced longitudinal cognitive decline [25]. Supporting this, an association between NLR and decreased CSF levels of Aβ, typically reflective of increased Aβ binding in the brain, has also been reported. Additionally, elevated NLR was associated with lower scores in global cognition, memory, and executive function, as well as with reduced brain metabolism and greater brain atrophy [92]. Similar results were reported in a study including 1579 participants of Chinese Han ethnicity diagnosed with a neurodegenerative condition, including AD, MCI, frontotemporal dementia, multiple system atrophy, progressive supranuclear palsy, Parkinson’s disease, motor neuron disease, and dementia with Lewy bodies. Higher NLR values, among other inflammatory indices, were associated with cognitive decline and neuropsychiatric disturbances (such as anxiety and depression), mediated by lower CSF Aβ levels and brain atrophy [94].

Such studies reinforce the hypothesis that innate immune dysregulation may be involved in the development of pathological changes closely related to the neurodegenerative processes typical of AD, or may serve as a potential risk factor for its onset.

All discussed studies are summarized in Table 1.

## 4. Discussion

Neuroinflammation is considered a key driver in neurodegenerative disease, and it has been largely investigated in AD [96]. CNS has been considered an immune-privileged organ for a long period due to the presence of the BBB. The BBB is a finely regulated structure supported by endothelial cells, astrocytes, and pericytes, and its main role is to protect the CNS from harmful pathogens. In addition, the BBB restricts the entry of most immune cells into the CNS, at least under physiological conditions [97]. Peripheral inflammation involves the activation of immune cells, including (i) neutrophils, the first responders of the innate immune system, which release enzymes and ROS; (ii) lymphocytes (T cells and B cells), representing the adaptive immune system and are involved in targeted inflammatory responses; (iii) monocytes, which can cross the BBB and differentiate into pro-inflammatory macrophages; and (iv) platelets, which contribute to inflammation by releasing cytokines. During chronic peripheral inflammation, these cells and their inflammatory mediators, including TNF-α, IL-1, IL-6, IL-17, and others, circulate in high amounts. When inflammation persists, the BBB can be affected, particularly its permeability. Disruption of the BBB is associated with the infiltration of immune cells, increased expression of adhesion molecules (such as ICAM-1), and activation of endothelial cells, which in turn promotes further cellular infiltration and amplifies inflammation [98]. Notably, neutrophils and T cells can infiltrate the CNS and contribute directly to neuronal damage and synaptic alterations. Neutrophils release ROS and other toxic species, while T cells may recognize and react against specific CNS antigens, further amplifying neuroinflammation [99]. This process activates microglia, the brain’s resident immune cells, which then release additional pro-inflammatory cytokines, creating a self-perpetuating pro-inflammatory environment. This leads to oxidative stress, neuronal injury, and synaptic loss, a common pathological pathway in neurodegenerative diseases [100]. In AD, these inflammatory and immune processes have been associated with increased Aβ and tau deposition, contributing to the neurodegenerative cascade [101]. These findings are supported by recent advances in MRI-based BBB imaging and microstructural MRI markers that assess BBB permeability and neuroinflammatory changes, allowing the identification of AD-related changes even at extremely early stages, thus enabling an early diagnosis [102]. MRI techniques allow the identification of the relationship between AD pathophysiology and BBB dysfunction, which occurs at a very early stage within the AD continuum [103]. In particular, BBB disruption, both through loss of tight junctions and aberrant AQP4 expression, leads to the accumulation of toxic substances (e.g., iron) that contribute to the toxic environment, promoting ROS production and increasing neuroinflammation [104].

The current review highlights, as the most consistently reported finding in the literature, that the NLR is elevated in individuals with AD, is correlated with the severity of cognitive decline, and may serve not only to distinguish individuals with MCI from HC, but also to identify those at higher risk of conversion to dementia. These findings have important implications for the field of neurodegenerative disease research, particularly in the context of clinical trials and pharmacological interventions.

The introduction of biomarker-based diagnostic criteria for AD has led to a profound conceptual shift, moving from a clinical to a biological definition of dementia [105]. Although revised in subsequent years, during which renewed emphasis was placed on the clinical-biological correlation [106], the “ATN framework”, based on markers of Aβ, tau, and neurodegeneration, remains central to the etiological classification of the various forms of dementia. Over the past decades, the search for biomarkers has primarily relied on imaging techniques, particularly PET, and on CSF analysis, methods that are relatively expensive or invasive. For this reason, recent research has focused on the validation of plasma biomarkers, starting with those specific to AD pathology [107]. Plasma is an accessible, economic and less invasive biofluid for detecting further potential biomarkers, and in this context NLR may represent an important tool to identify Patients with AD and stratify MCI subjects according to the risk of converting to dementia.

**Figure 2:**
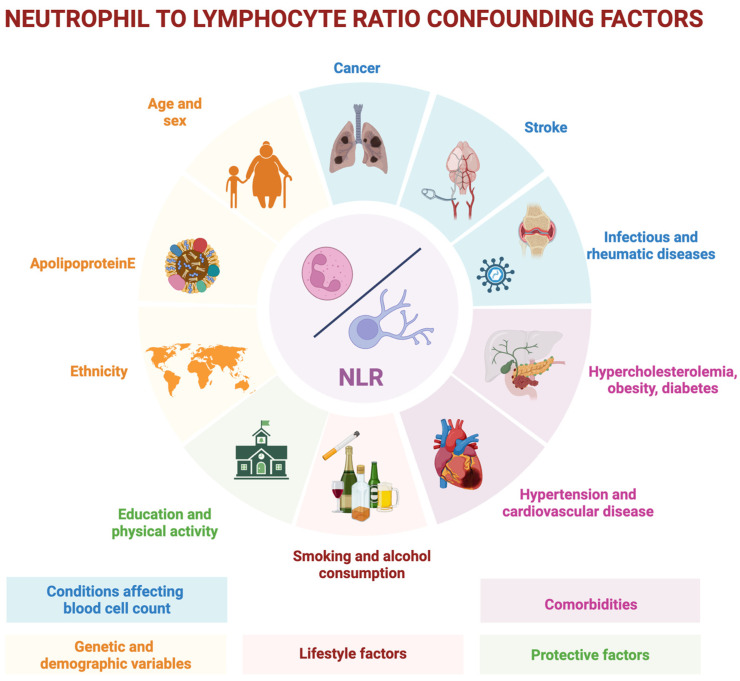
Key confounding factors influencing the value of NLR in cognitive decline. The figure highlights the main confounding factors that can affect NLR levels and impact cognitive decline studies. These include demographic, genetic, medical, lifestyle, and protective brain health variables, each with distinct mechanisms potentially influencing both NLR and neurodegeneration. NLR: neutrophil-to-lymphocyte ratio. Created with Biorender.com (De Marchi F.).

Further longitudinal, large-cohort studies are needed to confirm that peripheral inflammatory indices may serve as valuable and accessible biomarkers of early alterations occurring in AD. The NLR appears to be associated with pathological changes (such as Aβ brain deposition), neurodegeneration (such as hypometabolism or brain atrophy) and, consequently, with a more rapid cognitive decline or disease progression [25,93,94]. However, the clinical use of NLR presents several limitations. First, it is highly susceptible to the influence of underlying conditions, such as systemic inflammatory states, as well as the presence of infections, cancer, metabolic disorders, or vascular diseases [22,55]. In addition to comorbidities, numerous other variables, such as sex, genotype, and age, may also affect its values. Age itself is associated with changes in peripheral immune system functioning and ageing is associated with a low-grade systemic inflammation—the term “inflammaging” indicates this dysregulation [108]. This dysregulation can be linked to a higher production of ROS by senescent cells, neutrophil activations, cell apoptosis mechanisms failure [109] and it is reflected in altered peripheral blood cell counts. These factors significantly limit the diagnostic value of NLR, especially considering that reliable and disease-specific biomarkers of pathology and neurodegeneration are already available for AD. Since the NLR can be influenced by numerous factors, recent studies investigating its possible impact on cognitive decline have accounted for various confounding variables in their statistical analyses to ensure greater scientific rigor. These confounding factors may also help explain the wide variability observed in the findings of studies exploring the role of NLR in cognitive decline. The most commonly considered confounders across studies include genetic and demographic variables (such as age, sex, ethnicity, and APOE status), comorbidities (including diabetes, hypertension, hypercholesterolemia, cardiovascular disease, and obesity), conditions affecting blood cell counts (such as infectious diseases, rheumatic immune diseases, cancer, and stroke), lifestyle factors (such as smoking and alcohol consumption), and protective factors associated with brain health (such as education and physical activity). Figure 2 summarizes the main NLR confounding factors.

Ultimately, the most compelling aspect remains the prognostic value of this marker. NLR has proven to be a reliable prognostic index in various conditions, such as cancer [55], and in neurological disorders, including stroke [110,111]. It has also been associated with faster disease progression in neurodegenerative conditions [51,112,113]. In the context of neurodegenerative disease, the best opportunity to influence the progression of the pathological process is to identify individuals who show disease-specific brain changes and other alterations before any clear clinical symptoms appear [114]. Therefore, in AD, and in particular in the context of MCI or even in the preclinical dementia phase, the key value of NLR may lie in the ability to identify individuals at higher risk of progression, using a rapid and easily accessible test such as blood test. Nevertheless, the NLR needs to be accompanied by the use of diagnostic tools such as Aβ and tau and could find its place near other prognostic markers such as neurofilament light chain (NfL) in CSF. Aβ42 and tau proteins in CSF are validated biomarkers of AD and are integral to the ATN framework, which serves not only diagnostic and classification purposes but also has significant prognostic implications, since individuals with MCI who are positive for all markers show the highest likelihood of progression to dementia [115]. In this context, the NLR would position itself more as a purely prognostic marker, similar to NfL, reflecting neurodegeneration severity and progression risk, but without the direct pathophysiological specificity provided by Aβ or tau markers.

All considered, NLR could realistically be incorporated as a supplementary, easily accessible marker in cognitive assessments, but with cautious interpretation due to its non-specific nature. Integration into routine blood panels, combined with multi-modal biomarker frameworks, could make NLR a useful tool for stratifying prognosis. However, standardized cutoffs and confounding factor checklists are essential for meaningful clinical use.

## 5. Conclusions

Peripheral inflammation plays a significant role in the pathogenesis of AD, contributing to BBB disruption, neuroinflammation, and downstream neurodegeneration. The NLR, a simple and widely accessible blood-based marker, reflects this peripheral immune imbalance and has been consistently found to be elevated in individuals with AD. Although its diagnostic accuracy is limited by susceptibility to various confounding factors, NLR shows promise as a prognostic marker, particularly in identifying individuals with MCI who are at higher risk of progressing to dementia. Given its affordability, non-invasiveness, and potential link to AD-related pathology and neurodegeneration, NLR may be useful in research settings, especially for stratifying participants in clinical trials and studying early disease mechanisms. However, further large-scale, longitudinal studies are essential to validate its clinical relevance, refine its cut-off values, and clarify its role in the early identification and monitoring of AD progression.

## Figures and Tables

**Table 1:** Association between neutrophil-to-lymphocyte ratio and cognitive decline: key studies.

First Author, Year (Chronological Order)	Number of Included Participants	Main Results
Kuyumcu, M.E., 2012 [69]	241 AD, 175 HC	Higher NLR in AD vs. HC
Rembach, A., 2014 [70]	130 MCI, 205 AD, 759 HC	Higher NLR in AD vs. HC (if not corrected for age, sex, and APOEε4); NLR stable over disease course
Kalelioglu, T., 2017 [71]	31 AD, 30 MCI, 31 SCD, 31 HC	Higher NLR in AD and MCI vs. SCD and HC
Dong, X., 2019 [72]	56 AD, 57 MCI, 59 HC	Higher NLR in AD vs. HC; no differences between AD and MCI
An, P., 2019 [86]	186 MCI, 153 HC	Higher NLR in MCI vs. HC
Ramos-Cejudo, J., 2021 [89]	1684, of whom 51 had dementia (41 AD)	Individuals with higher NLR were at a greater risk of dementia
Kara, S.P., 2022 [78]	94 AD, 61 HC (and 100 PD)	Higher NLR in PD vs. HC and AD; no difference between AD and HC
Zhang, Y., 2022 [84]	361,653, of whom 4239 had dementia	Increased neutrophils, NLR, and SII were associated with higher dementia risk
Schröder, S., 2022 [77]	77 with dementia (of whom 33 had AD), 20 HC	No difference between different causes of dementia and HC
Hou, J., 2022 [92]	1107 AD	Elevated neutrophils and NLR were associated with lower global cognition, reduced brain metabolism by FDG-PET, and greater ventricular volume
Evlice, A., 2023 [73]	132 AD, 38 HC	Higher NLR in AD vs. HC
Li, J.Q., 2023 [94]	1579, of whom 440 AD	Higher neutrophils, monocytes, NLR, SII, PLR, and LMR were associated with cognitive decline
Tondo, G., 2023 [83]	130 MCI	Higher NLR, PLR, and SII in MCI converters to dementia
Yu, Z.W., 2023 [87]	376 MCI with diabetes, 441 only with diabetes	Elevated NLR is associated with MCI in patients with diabetes
Chou O, 2023 [88]	9760 (of whom 529 developed AD, 56 other dementias)	Higher NLR was associated with risk of development (no other dementia)
Zhuo, 2023 [93]	20,381 participants	SII and NLR were associated with general cognitive function and with the grey matter volume
Giannelli, 2023 [75]	51 MCI, 84 AD, 45 HC	Higher NLR in MCI and AD vs. HC
Algul, F., 2024 [74]	175 AD, 61 HC	Higher NLR and PLR in AD vs. HC
Cervellati, C., 2024 [76]	103 AD, 212 MCI, 34 VAD, 61 HC	NLR was higher in MCI or AD compared to VAD and HC
Jacobs, T., 2024 [95]	201 participants without cognitive impairment (from ADNI and NYU)	Associations between NLR and Aβ42 in the older ADNI cohort, and NLR and t-tau and p-tau in younger NYU cohort
Wang, 2025 [91]	2375 participants without cognitive impairment and 838 with dementia	Elevated NLR is elevated in demented and associated with increased risk of cognitive impairment

Summary of studies investigating the relationship between the neutrophil-to-lymphocyte ratio (NLR) and cognitive impairment, including Alzheimer’s disease (AD), mild cognitive impairment (MCI), and other dementias. The table presents the number of included participants and key findings for each study. Abbreviations: HC = healthy controls, SCD = subjective cognitive decline, PD = Parkinson’s disease, SII = systemic immune-inflammation index, PLR = platelet-to-lymphocyte ratio, LMR = lymphocyte-to-monocyte ratio, VAD = vascular dementia, Aβ42 = amyloid-beta 42, t-tau = total tau, p-tau = phosphorylated tau.

## References

[1] GBD 2019 Dementia Forecasting Collaborators (2022). Estimation of the global prevalence of dementia in 2019 and forecasted prevalence in 2050: An analysis for the Global Burden of Disease Study 2019. Lancet Public Health.

[2] Chen S., Cao Z., Nandi A., Counts N., Jiao L., Prettner K., Kuhn M., Seligman B., Tortorice D., Vigo D. (2024). The global macroeconomic burden of Alzheimer’s disease and other dementias: Estimates and projections for 152 countries or territories. Lancet Glob. Health.

[3] Bradfield N.I. (2023). Mild cognitive impairment: Diagnosis and subtypes. Clin. EEG Neurosci..

[4] He C.Y.Y., Zhou Z., Kan M.M.P., Chan D.H.Y., Wong A.C.T., Mok K.H.Y., Lam F.M.H., Chan S.C.C., Cheung C.K.C., Yeung M.K.C. (2024). Modifiable risk factors for mild cognitive impairment among cognitively normal community-dwelling older adults: A systematic review and meta-analysis. Ageing Res. Rev..

[5] Cummings J., Zhou Y., Lee G., Zhong K., Fonseca J., Cheng F. (2024). Alzheimer’s disease drug development pipeline: 2024. Alzheimer’s Dement. Transl. Res. Clin. Interv..

[6] Vickers J.C., Mitew S., Woodhouse A., Fernandez-Martos C.M., Kirkcaldie M.T., Canty A.J., McCormack G.H., King A.E. (2016). Defining the earliest pathological changes of Alzheimer’s disease. Curr. Alzheimer Res..

[7] Rostagno A.A. (2022). Pathogenesis of Alzheimer’s disease. Int. J. Mol. Sci..

[8] Tondo G., De Marchi F., Bonardi F., Menegon F., Verrini G., Aprile D., Anselmi M., Mazzini L., Comi C. (2024). Novel therapeutic strategies in Alzheimer’s disease: Pitfalls and challenges of anti-amyloid therapies and beyond. J. Clin. Med..

[9] Comi C., Tondo G. (2017). Insights into the protective role of immunity in neurodegenerative disease. Neural Regen. Res..

[10] Rajmohan R., Reddy P.H. (2017). Amyloid-beta and phosphorylated tau accumulations cause abnormalities at synapses of Alzheimer’s disease neurons. J. Alzheimer’s Dis..

[11] Kim M.E., Lee J.S. (2024). Mechanisms and Emerging Regulators of Neuroinflammation: Exploring New Therapeutic Strategies for Neurological Disorders. Curr. Issues Mol. Biol..

[12] Taipa R., das Neves S.P., Sousa A.L., Fernandes J., Pinto C., Correia A.P., Santos E., Pinto P.S., Carneiro P., Costa P. (2019). Proinflammatory and anti-inflammatory cytokines in the CSF of patients with Alzheimer’s disease and their correlation with cognitive decline. Neurobiol. Aging.

[13] Petersen K., Nallapu B., Lipton R., Ezzati A. (2024). CSF levels of proinflammatory and anti-inflammatory cytokines as predictors of cognitive decline across Alzheimer’s disease spectrum (N4. 004). Neurology.

[14] Gouilly D., Saint-Aubert L., Ribeiro M.J., Salabert A.S., Tauber C., Péran P., Arlicot N., Pariente J., Payoux P. (2022). Neuroinflammation PET imaging of the translocator protein (TSPO) in Alzheimer’s disease: An update. Eur. J. Neurosci..

[15] Rossano S.M., Johnson A.S., Smith A., Ziaggi G., Roetman A., Guzman D., Okafor A., Klein J., Tomljanovic Z., Stern Y. (2024). Microglia measured by TSPO PET are associated with Alzheimer’s disease pathology and mediate key steps in a disease progression model. Alzheimer’s Dement..

[16] Tondo G., Boccalini C., Caminiti S.P., Presotto L., Filippi M., Magnani G., Frisoni G.B., Iannaccone S., Perani D. (2021). Brain metabolism and microglia activation in mild cognitive impairment: A combined [18F] FDG and [11C]-(R)-PK11195 PET study. J. Alzheimer’s Dis..

[17] Hamelin L., Lagarde J., Dorothée G., Leroy C., Labit M., Comley R.A., de Souza L.C., Corne H., Dauphinot L., Bertoux M. (2016). Early and protective microglial activation in Alzheimer’s disease: A prospective study using 18 F-DPA-714 PET imaging. Brain.

[18] Fan Z., Brooks D.J., Okello A., Edison P. (2017). An early and late peak in microglial activation in Alzheimer’s disease trajectory. Brain.

[19] Guzman-Martinez L., Maccioni R.B., Andrade V., Navarrete L.P., Pastor M.G., Ramos-Escobar N. (2019). Neuroinflammation as a common feature of neurodegenerative disorders. Front. Pharmacol..

[20] Gao C., Jiang J., Tan Y., Chen S. (2023). Microglia in neurodegenerative diseases: Mechanism and potential therapeutic targets. Signal Transduct. Target. Ther..

[21] Verghese J.P., Terry A., de Natale E.R., Politis M. (2022). Research evidence of the role of the glymphatic system and its potential pharmacological modulation in neurodegenerative diseases. J. Clin. Med..

[22] Buonacera A., Stancanelli B., Colaci M., Malatino L. (2022). Neutrophil to lymphocyte ratio: An emerging marker of the relationships between the immune system and diseases. Int. J. Mol. Sci..

[23] Zahorec R. (2001). Ratio of neutrophil to lymphocyte counts--rapid and simple parameter of systemic inflammation and stress in critically ill. Bratisl. Lek. Listy.

[24] Aries M.L., Hensley-McBain T. (2023). Neutrophils as a potential therapeutic target in Alzheimer’s disease. Front. Immunol..

[25] Mehta N.H., Zhou L., Li Y., McIntire L.B., Nordvig A., Butler T., de Leon M., Chiang G.C. (2023). Peripheral immune cell imbalance is associated with cortical beta-amyloid deposition and longitudinal cognitive decline. Sci. Rep..

[26] Tseng C.W., Liu G.Y. (2014). Expanding roles of neutrophils in aging hosts. Curr. Opin. Immunol..

[27] Wigerblad G., Kaplan M.J. (2023). Neutrophil extracellular traps in systemic autoimmune and autoinflammatory diseases. Nat. Rev. Immunol..

[28] Yokoyama M., Kobayashi H., Tatsumi L., Tomita T. (2022). Mouse models of Alzheimer’s disease. Front. Mol. Neurosci..

[29] Webster S.J., Bachstetter A.D., Nelson P.T., Schmitt F.A., Van Eldik L.J. (2014). Using mice to model Alzheimer’s dementia: An overview of the clinical disease and the preclinical behavioral changes in 10 mouse models. Front. Genet..

[30] Cruz Hernández J.C., Bracko O., Kersbergen C.J., Muse V., Haft-Javaherian M., Berg M., Park L., Vinarcsik L.K., Ivasyk I., Rivera D.A. (2019). Neutrophil adhesion in brain capillaries reduces cortical blood flow and impairs memory function in Alzheimer’s disease mouse models. Nat. Neurosci..

[31] Baik S.H., Cha M.Y., Hyun Y.M., Cho H., Hamza B., Kim D.K., Han S.H., Choi H., Kim K.H., Moon M. (2014). Migration of neutrophils targeting amyloid plaques in Alzheimer’s disease mouse model. Neurobiol. Aging.

[32] Kong Y., Liu K., Hua T., Zhang C., Sun B., Guan Y. (2020). PET imaging of neutrophils infiltration in Alzheimer’s disease transgenic mice. Front. Neurol..

[33] Smyth L.C.D., Murray H.C., Hill M., van Leeuwen E., Highet B., Magon N.J., Osanlouy M., Mathiesen S.N., Mockett B., Singh-Bains M.K. (2022). Neutrophil-vascular interactions drive myeloperoxidase accumulation in the brain in Alzheimer’s disease. Acta Neuropathol. Commun..

[34] Volkman R., Ben-Zur T., Kahana A., Garty B.Z., Offen D. (2019). Myeloperoxidase deficiency inhibits cognitive decline in the 5XFAD mouse model of Alzheimer’s disease. Front. Neurosci..

[35] Song L., Yang Y.T., Guo Q., Zhao X., ZIB Consortium (2022). M. Cellular transcriptional alterations of peripheral blood in Alzheimer’s disease. BMC Med..

[36] Varathan P., Gorijala P., Jacobson T., Chasioti D., Nho K., Risacher S.L., Saykin A.J., Yan J. (2022). Integrative analysis of eQTL and GWAS summary statistics reveals transcriptomic alteration in Alzheimer brains. BMC Med. Genom..

[37] Zenaro E., Pietronigro E., Della Bianca V., Piacentino G., Marongiu L., Budui S., Turano E., Rossi B., Angiari S., Dusi S. (2015). Neutrophils promote Alzheimer’s disease–like pathology and cognitive decline via LFA-1 integrin. Nat. Med..

[38] Bowman G.L., Dayon L., Kirkland R., Wojcik J., Peyratout G., Severin I.C., Henry H., Oikonomidi A., Migliavacca E., Bacher M. (2018). Blood-brain barrier breakdown, neuroinflammation, and cognitive decline in older adults. Alzheimer’s Dement..

[39] Dong Y., Lagarde J., Xicota L., Corne H., Chantran Y., Chaigneau T., Crestani B., Bottlaender M., Potier M.C., Aucouturier P. (2018). Neutrophil hyperactivation correlates with Alzheimer’s disease progression. Ann. Neurol..

[40] Sakaguchi S., Mikami N., Wing J.B., Tanaka A., Ichiyama K., Ohkura N. (2020). Regulatory T cells and human disease. Annu. Rev. Immunol..

[41] Mietelska-Porowska A., Wojda U. (2017). T lymphocytes and inflammatory mediators in the interplay between brain and blood in Alzheimer’s disease: Potential pools of new biomarkers. J. Immunol. Res..

[42] Fu J., Huang Y., Bao T., Liu C., Liu X., Chen X. (2022). The role of Th17 cells/IL-17A in AD, PD, ALS and the strategic therapy targeting on IL-17A. J. Neuroinflamm..

[43] Hu D., Weiner H.L. (2024). Unraveling the dual nature of brain CD8+ T cells in Alzheimer’s disease. Mol. Neurodegener.

[44] Zhang Q., Yang G., Luo Y., Jiang L., Chi H., Tian G. (2024). Neuroinflammation in Alzheimer’s disease: Insights from peripheral immune cells. Immun. Ageing.

[45] Zhang J., Ke K.F., Liu Z., Qiu Y.H., Peng Y.P. (2013). Th17 cell-mediated neuroinflammation is involved in neurodegeneration of aβ1-42-induced Alzheimer’s disease model rats. PLoS ONE.

[46] Brigas H.C., Ribeiro M., Coelho J.E., Gomes R., Gomez-Murcia V., Carvalho K., Faivre E., Costa-Pereira S., Darrigues J., de Almeida A.A. (2021). IL-17 triggers the onset of cognitive and synaptic deficits in early stages of Alzheimer’s disease. Cell Rep..

[47] Cristiano C., Volpicelli F., Lippiello P., Buono B., Raucci F., Piccolo M., Iqbal A.J., Irace C., Miniaci M.C., Perrone Capano C. (2019). Neutralization of IL-17 rescues amyloid-β-induced neuroinflammation and memory impairment. Br. J. Pharmacol..

[48] Unger M.S., Li E., Scharnagl L., Poupardin R., Altendorfer B., Mrowetz H., Hutter-Paier B., Weiger T.M., Heneka M.T., Attems J. (2020). CD8+ T-cells infiltrate Alzheimer’s disease brains and regulate neuronal-and synapse-related gene expression in APP-PS1 transgenic mice. Brain Behav. Immun..

[49] Dansokho C., Ait Ahmed D., Aid S., Toly-Ndour C., Chaigneau T., Calle V., Cagnard N., Holzenberger M., Piaggio E., Aucouturier P. (2016). Regulatory T cells delay disease progression in Alzheimer-like pathology. Brain.

[50] Oberstein T.J., Taha L., Spitzer P., Hellstern J., Herrmann M., Kornhuber J., Maler J.M. (2018). Imbalance of circulating Th17 and regulatory T cells in Alzheimer’s disease: A case control study. Front. Immunol..

[51] Huang L.-T., Zhang C.-P., Wang Y.-B., Wang J.-H. (2022). Association of peripheral blood cell profile with Alzheimer’s disease: A meta-analysis. Front. Aging. Neurosci..

[52] Unger M.S., Marschallinger J., Kaindl J., Klein B., Johnson M., Khundakar A.A., Roßner S., Heneka M.T., Couillard-Despres S., Rockenstein E. (2018). Doublecortin expression in CD8+ T-cells and microglia at sites of amyloid-β plaques: A potential role in shaping plaque pathology?. Alzheimer’s Dementia.

[53] Roldgaard M., Benfield T., Tingsgård S. (2024). Blood neutrophil to lymphocyte ratio is associated with 90-day mortality and 60-day readmission in Gram negative bacteremia: A multi-center cohort study. BMC Infect. Dis..

[54] Ohashi K., Nishito Y., Fukuda H., Sadahiro R., Yoshida Y., Watanabe S.I., Motoi N., Sonobe Y., Mizuno H., Tsunoda H. (2024). Neutrophil-to-lymphocyte ratio is a prognostic factor reflecting immune condition of tumor microenvironment in squamous cell lung cancer. Sci. Rep..

[55] Cupp M.A., Cariolou M., Tzoulaki I., Aune D., Evangelou E., Berlanga-Taylor A.J. (2020). Neutrophil to lymphocyte ratio and cancer prognosis: An umbrella review of systematic reviews and meta-analyses of observational studies. BMC Med..

[56] Lin L., Yang J., Fu W., Liu X., Liu Y., Zou L. (2024). Association between neutrophil-to-lymphocyte ratio and short-term all-cause mortality in patients with cerebrovascular disease admitted to the intensive care unit-a study based on the MIMIC-IV database. Front. Med..

[57] Xue J., Huang W., Chen X., Li Q., Cai Z., Yu T., Shao B. (2017). Neutrophil-to-lymphocyte ratio is a prognostic marker in acute ischemic stroke. J. Stroke Cerebrovasc. Dis..

[58] Fridlender Z.G., Albelda S.M. (2012). Tumor-associated neutrophils: Friend or foe?. Carcinogenesis.

[59] Heshmat-Ghahdarijani K., Sarmadi V., Heidari A., Falahati Marvasti A., Neshat S., Raeisi S. (2023). The neutrophil-to-lymphocyte ratio as a new prognostic factor in cancers: A narrative review. Front. Oncol..

[60] DeMaio A., Mehrotra S., Sambamurti K., Husain S. (2022). The role of the adaptive immune system and T cell dysfunction in neurodegenerative diseases. J. Neuroinflamm..

[61] Mohammadi A., Mohammadi M., Almasi-Dooghaee M., Mirmosayyeb O. (2024). Neutrophil to lymphocyte ratio in Alzheimer’s disease: A systematic review and meta-analysis. PLoS ONE.

[62] Grillo P., Sancesario G.M., Bovenzi R., Zenuni H., Bissacco J., Mascioli D., Simonetta C., Forti P., Degoli G.R., Pieri M. (2023). Neutrophil-to-lymphocyte ratio and lymphocyte count reflect alterations in central neurodegeneration-associated proteins and clinical severity in Parkinson Disease patients. Parkinsonism Relat. Disord..

[63] Muñoz-Delgado L., Macías-García D., Jesús S., Martín-Rodríguez J.F., Labrador-Espinosa M.Á., Jiménez-Jaraba M.V., Adarmes-Gómez A., Carrillo F., Mir P. (2021). Peripheral immune profile and neutrophil-to-lymphocyte ratio in Parkinson’s disease. Mov. Disord..

[64] Nona R.J., Henderson R.D., McCombe P.A. (2024). Neutrophil-to-lymphocyte ratio at diagnosis as a biomarker for survival of amyotrophic lateral sclerosis. Amyotroph. Lateral Scler. Front. Degener..

[65] Cotet C., Alarcan H., Hérault O., Corcia P., Vourc’h P., Andres C.R., Blasco H., Veyrat-Durebex C. (2023). Neutrophil to lymphocyte ratio as a prognostic marker in amyotrophic lateral sclerosis. Biomolecules.

[66] Grassano M., Manera U., De Marchi F., Cugnasco P., Matteoni E., Daviddi M., Solero L., Bombaci A., Palumbo F., Vasta R. (2023). The role of peripheral immunity in ALS: A population-based study. Ann. Clin. Transl. Neurol..

[67] Choi S.J., Hong Y.H., Kim S.M., Shin J.Y., Suh Y.J., Sung J.J. (2020). High neutrophil-to-lymphocyte ratio predicts short survival duration in amyotrophic lateral sclerosis. Sci. Rep..

[68] Hasselbalch I.C., Søndergaard H.B., Koch-Henriksen N., Olsson A., Ullum H., Sellebjerg F., Oturai A.B. (2018). The neutrophil-to-lymphocyte ratio is associated with multiple sclerosis. Multiple Sclerosis J.–Exp. Transl. Clin..

[69] Kuyumcu M.E., Yesil Y., Oztürk Z.A., Kizilarslanoğlu C., Etgül S., Halil M., Ulger Z., Cankurtaran M., Arıoğul S. (2012). The evaluation of neutrophil-lymphocyte ratio in Alzheimer’s disease. Dement. Geriatr. Cogn. Disord..

[70] Rembach A., Watt A.D., Wilson W.J., Rainey-Smith S., Ellis K.A., Rowe C.C., Villemagne V.L., Macaulay S.L., Bush A.I., Martins R.N. (2014). An increased neutrophil–lymphocyte ratio in Alzheimer’s disease is a function of age and is weakly correlated with neocortical amyloid accumulation. J. Neuroimmunol..

[71] Kalelioglu T., Yuruyen M., Gultekin G., Yavuzer H., Özturk Y., Kurt M., Topcu Y., Doventas A., Emul M. (2017). The neutrophil and platelet to lymphocyte ratios in people with subjective, mild cognitive impairment and early Alzheimer’s disease. Eur. Psychiatry.

[72] Dong X., Nao J., Shi J., Zheng D. (2019). Predictive value of routine peripheral blood biomarkers in Alzheimer’s disease. Front. Aging Neurosci..

[73] Evlice A., Sanli Z.S., Boz P.B. (2023). The importance of Vitamin-D and neutrophil-lymphocyte ratio for Alzheimer’s Disease. Pak. J. Med. Sci..

[74] Algul F.E., Kaplan Y. (2024). Increased Systemic Immune-Inflammation Index as a Novel Indicator of Alzheimer’s Disease Severity. J. Geriatr. Psychiatry Neurol..

[75] Giannelli R., Canale P., Del Carratore R., Falleni A., Bernardeschi M., Forini F., Biagi E., Curzio O., Bongioanni P. (2023). Ultrastructural and molecular investigation on peripheral leukocytes in Alzheimer’s disease patients. Int. J. Mol. Sci..

[76] Cervellati C., Pedrini D., Pirro P., Guindani P., Renzini C., Brombo G., Zuliani G. (2024). Neutrophil–Lymphocytes Ratio as Potential Early Marker for Alzheimer’s Disease. Mediators Inflamm..

[77] Schröder S., Heck J., Groh A., Frieling H., Bleich S., Kahl K.G., Bosch J.J., Krichevsky B., Schulze-Westhoff M. (2022). White blood cell and platelet counts are not suitable as biomarkers in the differential diagnostics of dementia. Brain Sci..

[78] Kara S.P., Altunan B., Unal A. (2022). Investigation of the peripheral inflammation (neutrophil–lymphocyte ratio) in two neurodegenerative diseases of the central nervous system. Neurol. Sci..

[79] Duara R., Barker W. (2022). Heterogeneity in Alzheimer’s disease diagnosis and progression rates: Implications for therapeutic trials. Neurotherapeutics.

[80] Menegon F., De Marchi F., Aprile D., Zanelli I., Decaroli G., Comi C., Tondo G. (2024). From mild cognitive impairment to dementia: The impact of comorbid conditions on disease conversion. Biomedicines.

[81] Chen J.Y., Liang S.K., Chuang T.Y., Chu C.Y., Tu C.H., Yeh Y.J., Wei Y.F., Chen K.Y. (2024). The impact of comorbidities, neutrophil-to-lymphocyte ratio, and drug toxicities on quality of life in lung cancer patients receiving EGFR-TKI therapy. J. Formos. Med. Assoc..

[82] Hung K.C., Liu C.C., Wu J.Y., Ho C.N., Lin M.C., Hsing C.H., Chen I.W. (2023). Association between the neutrophil-to-lymphocyte ratio and cognitive impairment: A meta-analysis of observational studies. Front. Endocrinol..

[83] Tondo G., Aprile D., De Marchi F., Sarasso B., Serra P., Borasio G., Rojo E., Arenillas J.F., Comi C. (2023). Investigating the prognostic role of peripheral inflammatory markers in mild cognitive impairment. J. Clin. Med..

[84] Zhang Y.R., Wang J.J., Chen S.F., Wang H.F., Li Y.Z., Ou Y.N., Huang S.Y., Chen S.D., Cheng W., Feng J.F. (2022). Peripheral immunity is associated with the risk of incident dementia. Mol. Psychiatry.

[85] Irwin K., Sexton C., Daniel T., Lawlor B., Naci L. (2018). Healthy aging and dementia: Two roads diverging in midlife?. Front. Aging Neurosci..

[86] An P., Zhou X., Du Y., Zhao J., Song A., Liu H., Ma F., Huang G. (2019). Association of neutrophil-lymphocyte ratio with mild cognitive impairment in elderly Chinese adults: A case-control study. Curr. Alzheimer Res..

[87] Yu Z.W., Wang Y., Li X., Tong X.W., Zhang Y.T., Gao X.Y. (2023). Association between the neutrophil to lymphocyte ratio and mild cognitive impairment in patients with type 2 diabetes. Aging Clin. Exp. Res..

[88] Chou O.H.I., Zhou J., Li L., Chan J.S.K., Satti D.I., Chou V.H.C., Wong W.T., Lee S., Cheung B.M.Y., Tse G. (2023). The association between neutrophil-lymphocyte ratio and variability with new-onset dementia: A population-based cohort study. J. Alzheimer’s Dis..

[89] Ramos-Cejudo J., Johnson A.D., Beiser A., Seshadri S., Salinas J., Berger J.S., Fillmore N.R., Do N., Zheng C., Kovbasyuk Z. (2021). The neutrophil to lymphocyte ratio is associated with the risk of subsequent dementia in the framingham heart study. Front. Aging Neurosci..

[90] van der Willik K.D., Fani L., Rizopoulos D., Licher S., Fest J., Schagen S.B., Ikram M.K., Ikram M.A. (2019). Balance between innate versus adaptive immune system and the risk of dementia: A population-based cohort study. J. Neuroinflamm..

[91] Wang X., Wang B., Du X., Liu P., Yang F., Su J., Zhang Y. (2025). Associations between neutrophil–lymphocyte ratio and risk of cognitive impairment among Chinese older adults. BMC Geriatr..

[92] Hou J.H., Ou Y.N., Xu W., Zhang P.F., Tan L., Yu J.T., Alzheimer’s Disease Neuroimaging Initiative (2022). Association of peripheral immunity with cognition, neuroimaging, and Alzheimer’s pathology. Alzheimers Res. Ther..

[93] Zhuo B., Zheng D., Cai M., Wang C., Zhang S., Zhang Z., Tian F., Wang X., Lin H. (2023). Mediation effect of brain volume on the relationship between peripheral inflammation and cognitive decline. J. Alzheimer’s Dis..

[94] Li J.Q., Zhang Y.R., Wang H.F., Guo Y., Shen X.N., Li M.M., Song J.H., Tan L., Xie A.M., Yu J.T. (2023). Exploring the links among peripheral immunity, biomarkers, cognition, and neuroimaging in Alzheimer’s disease. Alzheimer’s Dement. Diagn. Assess. Dis. Monit..

[95] Jacobs T., Jacobson S.R., Fortea J., Berger J.S., Vedvyas A., Marsh K., He T., Gutierrez-Jimenez E., Fillmore N.R., Gonzalez M. (2024). The neutrophil to lymphocyte ratio associates with markers of Alzheimer’s disease pathology in cognitively unimpaired elderly people. Immun. Ageing.

[96] Heneka M.T., van der Flier W.M., Jessen F., Hoozemanns J., Thal D.R., Boche D., Brosseron F., Teunissen C., Zetterberg H., Jacobs A.H. (2024). Neuroinflammation in Alzheimer disease. Nat. Rev. Immunol..

[97] Kadry H., Noorani B., Cucullo L. (2020). A blood–brain barrier overview on structure, function, impairment, and biomarkers of integrity. Fluids Barriers CNS.

[98] Marchetti L., Engelhardt B. (2020). Immune cell trafficking across the blood-brain barrier in the absence and presence of neuroinflammation. Vasc. Biol..

[99] Kanashiro A., Hiroki C.H., da Fonseca D.M., Birbrair A., Ferreira R.G., Bassi G.S., Fonseca M.D., Kusuda R., Cebinelli G.C.M., da Silva K.P. (2020). The role of neutrophils in neuro-immune modulation. Pharmacol. Res..

[100] Dash U.C., Bhol N.K., Swain S.K., Samal R.R., Nayak P.K., Raina V., Panda S.K., Kerry R.G., Duttaroy A.K., Jena A.B. (2025). Oxidative stress and inflammation in the pathogenesis of neurological disorders: Mechanisms and implications. Acta Pharm. Sin. B.

[101] Balkhi S., Di Spirito A., Poggi A., Mortara L. (2025). Immune Modulation in Alzheimer’s Disease: From Pathogenesis to Immunotherapy. Cells.

[102] Uchida Y., Hou Z., Gomez-Isaza L., Luongo M., Troncoso J.C., Miller M.I., Mori S., Oishi K. (2025). Quantification of perforant path fibers for early detection of Alzheimer’s disease. Alzheimer’s Dement..

[103] Uchida Y., Kan H., Sakurai K., Oishi K., Matsukawa N. (2023). Contributions of blood–brain barrier imaging to neurovascular unit pathophysiology of Alzheimer’s disease and related dementias. Front. Aging Neurosci..

[104] Uchida Y., Kan H., Sakurai K., Horimoto Y., Hayashi E., Iida A., Okamura N., Oishi K., Matsukawa N. (2022). APOE ɛ4 dose associates with increased brain iron and β-amyloid via blood–brain barrier dysfunction. J. Neurol. Neurosurg. Psychiatry.

[105] Jack C.R., Bennett D.A., Blennow K., Carrillo M.C., Dunn B., Haeberlein S.B., Holtzman D.M., Jagust W., Jessen F., Karlawish J. (2018). NIA-AA research framework: Toward a biological definition of Alzheimer’s disease. Alzheimer’s Dement..

[106] Jack C.R., Andrews S.J., Beach T.G., Buracchio T., Dunn B., Graf A., Hansson O., Ho C., Jagust W., McDade E. (2024). Revised criteria for the diagnosis and staging of Alzheimer’s disease. Nat. Med..

[107] Pais M.V., Forlenza O.V., Diniz B.S. (2023). Plasma biomarkers of Alzheimer’s disease: A review of available assays, recent developments, and implications for clinical practice. J. Alzheimers Dis. Rep..

[108] Kosyreva A.M., Sentyabreva A.V., Tsvetkov I.S., Makarova O.V. (2022). Alzheimer’s disease and inflammaging. Brain Sci..

[109] Abbatecola A.M., Giuliani A., Biscetti L., Scisciola L., Battista P., Barbieri M., Sabbatinelli J., Olivieri F. (2024). Circulating biomarkers of inflammaging and Alzheimer’s disease to track age-related trajectories of dementia: Can we develop a clinically relevant composite combination?. Ageing Res. Rev..

[110] Li W., Hou M., Ding Z., Liu X., Shao Y., Li X. (2021). Prognostic value of neutrophil-to-lymphocyte ratio in stroke: A systematic review and meta-analysis. Front. Neurol..

[111] Ying Y., Yu F., Luo Y., Feng X., Liao D., Wei M., Li X., Huang Q., Liu Z., Zhang L. (2021). Neutrophil-to-lymphocyte ratio as a predictive biomarker for stroke severity and short-term prognosis in acute ischemic stroke with intracranial atherosclerotic stenosis. Front. Neurol..

[112] Li F., Weng G., Zhou H., Zhang W., Deng B., Luo Y., Tao X., Deng M., Guo H., Zhu S. (2024). The neutrophil-to-lymphocyte ratio, lymphocyte-to-monocyte ratio, and neutrophil-to-high-density-lipoprotein ratio are correlated with the severity of Parkinson’s disease. Front. Neurol..

[113] Huang W.C., Lin H.C., Yang Y.H., Hsu C.W., Chen N.C., Tsai W.C., Cheng B.C., Tsai N.W. (2022). Neutrophil-to-lymphocyte ratio and monocyte-to-lymphocyte ratio are associated with a 2-year relapse in patients with multiple sclerosis. Mult. Scler. Relat. Disord..

[114] Tondo G., De Marchi F. (2022). From biomarkers to precision medicine in neurodegenerative diseases: Where are we?. J. Clin. Med..

[115] Prestia A., Caroli A., van der Flier W.M., Ossenkoppele R., Van Berckel B., Barkhof F., Teunissen C.E., Wall A.E., Carter S.F., Schöll M. (2013). Prediction of dementia in MCI patients based on core diagnostic markers for Alzheimer disease. Neurology.

